# Medical Society of the County of Chautauqua

**Published:** 1896-02

**Authors:** C. A. Ellis

**Affiliations:** Secretary


### MEDICAL SOCIETY OF THE COUNTY OF CHAUTAUQUA.

*Semi-annual meeting, January 14, 1896.*

Reported by C. A. ELLIS, M. D., Secretary.

THE semi-annual meeting of the Medical Society of the County of Chautauqua was held at the Sherman house, Jamestown, Tuesday, January 14, 1896.

The meeting was called to order at 11 o'clock A. M. by the president, Dr. E. S. Rich, of Kennedy. There was a full attendance of members during the sessions.

The program was carried out as published in the January issue of the JOURNAL, except that the paper announced on Criminal abortion, by Dr. James Murphy, of Sherman, was not read, owing to the absence of the author.

Dr. A. D. LAKE, of Gowanda, addressed the society on the subject of Tuberculosis among the Indians on the Cattaraugus reservation. He offered resolutions inviting the State Board of Health to investigate the causes of tuberculosis among the Indians of the State of New York, suggesting the employment of a competent bacteriologist and pathologist to aid in the research. The resolutions were unanimously adopted.

The society next proceeded to consider the recent action of the Board of Supervisors in fixing the fee for the examinations of the insane at $3.00. Resolutions were unanimously passed condemning the action of the Supervisors and establishing the fee for such examination at $10.00, with 25 cents mileage, and declaring it to be dishonorable to perform the service at a lower rate.

A resolution was also passed declaring that the fee for post mortem examinations should be not less than $50.00.

The following named new members were admitted: Honorary—Rev. W. K. Crosby, Falconer. Active—Drs. R. B. Parks, W. H. Hotchkiss, J. M. Brooks and W. D. Wellman, Jamestown; Dr. J. R. Smith, Conewango Valley; and Dr. W. O. Smith, Falconer.

The society instructed the board of censors to vigorously prosecute all persons in the county who may be found engaged in the illegal practice of medicine, and then adjourned.

